# Practical Notes and Formulæ

**Published:** 1878-12-20

**Authors:** 


### *Practical Notes and Formulae.*

---

G. L. Glazener M. D. of Greenville, S. C., writes:

*Editor Medical Record:*

DEAR SIR:—In the treatment of slight wounds, cuts, abrasions, and on surfaces where the integument has been lost, and cannot well be replaced even by surgical operation. I know of nothing in my practice that has done so much good as the *Balsam of Fir.*

The article I use has been invariably obtained from the Balsam or Blue mountains of Western North Carolina. I usually make a plaster and cover the wound, and let it alone for four or six days, and generally have the pleasure of finding the wound healed. Generally there is no inflammatory condition, but if there should be these balsam plasters do not prevent the use of cold water applications. Last year a little boy 10 years old was brought into my office w th the entire posterior surface of the right hand removed by some of the machinery in Comperdown mills—the skin being entirely removed. After working all the blood away, I drew the parts as best I could towards each other with adhesive strips, and then covered with a plaster of Balsam Fir. No other dressing was ordered, the boy was not confined, but went at will, and on the 9th day I removed the dressing and found nearly the entire large surface healed by granulation. I put on another and less plaster, and in a few days he returned into the mills to his work.

Shortly after this I was called to see a child bitten by a dog. I found a three inch gash over the left eye—the wound gaping wide. I closed with three stitches and adhesive strips, and placed a plaster as above over these. Four days after I removed the stitches and found the gash entirely healed. A few strips were placed across to prevent accidental tearing, no appearance left except a long close cicatrix. These are only a few of the many cases in which I have used the Balsam of Fir. I have never found wounds dressed in this way to emit any bad odors, and old sores on legs will assume a healthier appearance.

---

### TREATMENT OF DYSPEPSIA.

---

The following is the treatment adopted at the Demilt Dispensary, New York, as described by Dr. D. Lewis, in the New York *Medical Journal:*

| | | |
|---|---|---|
| ℞. | Pulv. rhei., | ʒj |
| | Sodæ bicarb., | ʒiss |
| | Ol. menth. vir., | gtts. iv |
| | Aquæ, | ℥iv. M. |

SIG.—A tablespoonful before meals.

This akaline mixture probably owes its efficacy to its stimulating action upon the gastric glands—a property of alkalies which has been amply demonstrated by many experimenters. When an additional laxative was necessary, a compound rhubarb pill was ordered at bedtime, or, what is preferable in many cases, the pill of aloes, belladonna, and strychnia—

| | | |
|---|---|---|
| ℞. | Ext. aloes, | grs. ijss |
| | Ext. belladonnæ, | |
| | Ext. nucis vom., aa | gr. ¼. M. |

SIG.—One at bedtime.

In contrast with the above case are those patients who are anæmic, and complain of the symptoms common to that condition—loss of appetite, palpitation of the heart, intercostal neuralgia and headache. In some instances this condition is a natural sequence of prolonged dyspepsia, but is more commonly dependent upon other causes, such as bad hygiene, overwork, or malarial influences. Tonic treatment is here indicated, and the following prescription is usually effective:

℞. Quiniæ sulph., gr. xij

Tr. ferri chloridi, ʒiijss

Aquæ, ℥iv. M.

SIG.—A teaspoonful in a wineglass of cold water, half an hour after meals.

An aloes and belladonna pill is occasionally required at bedtime.

Plasters have been often prescribed for intercostal neuralgia in these cases. Notwithstanding the prejudice against their use, experience here has proved them to be a valuable adjuvant in the treatment.

The belladonna plaster (4x6) is the one most frequently ordered, and next in order the capsicum plaster (same size), as now kept by druggists. A pitch-plaster, with chloral hydrate sprinkled over its surface, was tried in several cases, but proved inferior to either of the others.

When there was irritability of the stomach (probably gastritis), with nausea and vomiting, a bismuth mixture was often ordered—

℞. Bismuth, subnit., ʒiv

Acid. nitric. dil., ʒiij

Tr. nucis vom., ʒjss

Aq. menth. pip., ℥iv. M.

SIG.—A teaspoonful after meals. Shake well before using.

Since it has been pretty clearly demonstrated that bismuth acts mechanically by adhering to the mucous coat of the stomach, it is evident that a large dose should be administered. But the *very large* doses given by Lusanne, Menneret, and others (who gave ℥j per diem), no doubt hinder the excretion of gastric juice, thereby causing the cachetic symptoms which those observers found to follow its prolonged use.

---

CHLORAL HYDRATE LOCALLY APPLIED IN TETANUS.—Dr. Bigelow reports in the Practitioner a case of tetanus, caused by a rusty nail penetrating the foot, which was relieved in less than twenty minutes by introducing chloral hydrat. ʒ i into the wound after it had been enlarged by incision.—*Ohio Med. Recorder.*

---

### IMPOTENCE AND SPERMATORRHŒA.

Treatment of Impotence and Spermatorhœa. Dr Beard in addition to electricity, employs the following:

Internal treatment consisted in the use of gelsemium, bromide of camphor, ergotin, and lupulin, Gelsemium alone was of more value than was usually supposed. A pill made up somewhat as follows might be used :

R Gelsemium.... ........................ ½ gr.

Bromide camphor................ 1 ½grs.

Ergotin and lupulin aa........... ¾gr.

He sometimes used bromide of zinc and valerianate of zinc combined, aa gr. i., given in the form of pill, and gradually increasing the dose.

---

CHLORYMAL.—Dr. G. B. Sanford recommends in the N. Y. *Medical Record* the following combination as safer and more satisfactory than chloroform uncombined.

R. Squibbs chloroform.............. 1 pound
Nitrite of amyl................. 2 drachms

It is advised that the nitrite of amyl be diminished in long and tedious operations, and, if it be found necessary to vary the proportions, the point aimed at being to use just sufficient to counteract the paralytic effect of the chloroform.

---

The *Druggist Circular* gives the following as a cheap paint for rough woodwork or fences : Six pounds of melted pitch, one pint of linseed oil, and one pound of brick dust or yellow ochre. It is excellent and will stand for years.

---

*Iodoformised Cod Liver Oil.*—Dissolve one part of iodoform in 200 of cod liver oil, and add 0.5 of oil of ainseed. The dose is a tablespoonful twice or thrice daily in phthisis, glandular affections, and scrofula.

### URTICARIA, OR NETTLE RASH.

Urticaria is usually the result of gastric disturbance from indulgence in rich and overseasoned articles of diet, and will commonly disappear after the use of a purgative and the regulation of the diet. For thc intolerable itching that sometimes attends this disease, preventing sleep at night, a dose of quinine will be found useful. As a local application brandy or whiskey will usually give prompt relief.

---

BLUE BLACK WRTING INK.

| | | | | |
|---|---|---|---|---|
| B. | Alleppo galls, bruised....oz jx | ( 290 | 00) |
| | Bruieed cloves...........dr. ij | ( 8 | 00) |
| | Cold water........... oz lxxx | (2460 | 00) |
| | Sulphate of iron........oz. viij | ( 256 | 00) |
| | Sulphuric acid...........m lxx | ( 4 | 50) |
| | Indigo paste .............dr. jv | ( 16 | 00) |

Place the galls with the cloves in a gallon bottle, pour upon them the water, and digest, shaking often for a fortnight. Press and filter through paper into another gallon bottle. Next put in the sulphate of iron, dissolve it, add the acid and shake briskly. Lastly, add the indigo, mix well and filter through paper. The ink is to be kept in well corked bottles. The writing is at first pale green but it soon turns to deep jet black. It is not copying, but may be rendered such by the addition of sugar or glycerine.—*Druggists' Circular.*

---

PRESCRIPTION FOR AMENORRHŒA.—In amenorrhœa from anæmia and chlorosis:

℞. Pulv. Ferri. Sulphat., Potass. Carb. Puræ, aa ʒij; Mucil. Tragacanthi, q. s.; M.—Ft. in pill no xlviii.

S. To be given daily, in doses gradually increasing until three pills are taken after each meal.—*Hosp. Gazette.*

---

*Antirheumatic Pills* (Knoll).—Iodoform, reduced iron, each 3 grammes (46½ grains); purified liquorice juice, enough to make 60 pills, to be sprinkled with lycopodium. Two to be taken three times daily.

### BITTERS.

Wormwood......... 6 drachms.
Gentian root........12 "
Angelica root....... 1½ "
Galangal root....... 2½ "
Ginger............. 2½ "
Cinnamon.......... 4 "
Cloves.............45 grains.
Orange buds........20 drachms.
Oil lemon..........25 drops.
Oil star anise........20 "
Oil cassia...........30 "

To one gallon of whiskey.
Dose, one tablespoonful.

### TO KEEP FLIES FROM OPEN SORES ON DOMESTIC ANIMALS.

Oil wormwood........ 6 drops.
Olive oil.... .........12 drachms.
Carbolic acid.......... 8 drops.

Apply to the sores.

### OINTMENT FOR TETTA.

Beeswax............ 6 drachms.
Lard................ 2 ounces.
Carbolic acid........45 drops.

Add—

Peruvian balsam...... ½ drachm.

Mix.
Spread very thin.

### FOR FROSTED FEET AND HANDS.

Almond oil............ ½ ounce.

Beeswax.............. ½ drachm.

Spermaceti............ 1 "

Peruvian balsam......... ½ ounce.

Conc. muriatic acid......45 drops.

### CATARRH SNUFF.

(*Does not provoke sneezing*)

Tannin. .............15 grains.
Powdered rose leaves....25 drachms.
Sugar.. ......... ...25 "

Mix.

### PIMPLE CERATE.

Tannin...............75 grains.
Bitter almond water.. 1½ drachms.
Precipitated sulphur.... 1 drachm.
Lard................. 2 ounces.

Mix.
—*Druggists Circular.*

